# Effects of Text4Hope-Addiction Support Program on Cravings and Mental Health Symptoms: Results of a Longitudinal Cross-sectional Study

**DOI:** 10.2196/40440

**Published:** 2023-03-01

**Authors:** Gloria Obuobi-Donkor, Reham Shalaby, Wesley Vuong, Belinda Agyapong, Marianne Hrabok, April Gusnowski, Shireen Surood, Andrew J Greenshaw, Vincent IO Agyapong

**Affiliations:** 1 Department of Psychiatry, Faculty of Medicine, Dalhousie University Halifax, NS Canada; 2 Department of Psychiatry, University of Alberta Edmonton, AB Canada; 3 Alberta Health Services Edmonton, AB Canada; 4 Department of Psychiatry University of Calgary Calgary, AB Canada

**Keywords:** addiction, substance craving, depression, anxiety, Text4Hope, satisfaction

## Abstract

**Background:**

Drug misuse is complex, and various treatment modalities are emerging. Providing supportive text messages to individuals with substance use disorder offers the prospect of managing and improving symptoms of drug misuse and associated comorbidities.

**Objective:**

This study evaluated the impact of the daily supportive text message program (Text4Hope-Addiction Support) in mitigating cravings and mental health symptoms in subscribers and quantify user satisfaction with the Text4Hope-Addiction Support program.

**Methods:**

Subscribers to the Text4Hope-Addiction Support program received daily supportive text messages for 3 months; the messages were crafted based on addiction counseling and cognitive behavioral therapy principles. Participants completed an anonymous web-based questionnaire to assess cravings, anxiety, and depressive symptoms using the Brief Substance Craving Scale (BSCS), Generalized Anxiety Disorder-7 (GAD-7) scale, and Patient Health Questionnaire-9 (PHQ-9) scale at enrollment (baseline), after 6 weeks, and after 3 months. Likert scale satisfaction responses were used to assess various aspects of the Text4Hope-Addiction program.

**Results:**

In total, 408 people subscribed to the program, and 110 of 408 (26.9%) subscribers completed the surveys at least at one time point. There were significant differences between the mean baseline and 3-month BSCS scores *P*=.01 (−2.17, 95% CI –0.62 to 3.72), PHQ-9 scores, *P*=.004 (−5.08, 95% CI −1.65 to −8.51), and GAD-7 scores, *P*=.02 (−3.02, 95% CI −0.48 to −5.56). Participants who received the supportive text messages reported a reduced desire to use drugs and a longer time interval between substance use, which are reflected in 41.1% and 32.5% decrease, respectively, from baseline score. Approximately 89% (23/26) of the participants agreed that Text4Hope-Addiction program helped them cope with addiction-related stress, and 81% (21/25) of the participants reported that the messages assisted them in dealing with anxiety. Overall, 69% (18/26) of the participants agreed that it helped them cope with depression related to addiction; 85% (22/26) of the participants felt connected to a support system; 77% (20/26) of the participants were hopeful of their ability to manage addiction issues; and 73% (19/26) of the participants felt that their overall mental well-being was improved. Most of the participants agreed that the interventions were always positive and affirmative (19/26, 73%), and succinct (17/26, 65%). Furthermore, 88% (21/24) of the participants always read the messages; 83% (20/24) of the participants took positive or beneficial actions after reading; and no participant took a negative action after reading the messages. In addition, most participants agreed to recommend other diverse technology-based services as an adjunctive treatment for their mental and physical health disorders.

**Conclusions:**

Subscribers of Text4Hope-Addiction Support program experienced improved mental health and addiction symptoms. Addiction care practitioners and policy makers can implement supportive text-based strategies to complement conventional treatments for addiction, given that mobile devices are widely used.

## Introduction

### Background

Substance misuse and abuse and related comorbid mental health conditions are disabling global health concerns, requiring costly treatment modalities. The World Economic Forum projected that approximately US $16 trillion would be lost globally owing to substance misuse and mental health conditions in the next 20 years [1], with inadequate treatment options for substance use disorders [1,2]. The lifetime prevalence of substance use disorder in Canada is estimated at 21.6% [3], and US $30.7 billion was spent on treating substance use and misuse in 2002 [4,5]. Similarly, in the United States, the annual national bill for substance use disorder and its related effects is >US $740 billion, and approximately 900,000 people die of alcohol abuse [6-8]. Addiction services can be advantageous in reducing the complications of substance use disorders, but there are barriers to assessing these services. An examination of the rationale for delay and failure in the treatment of mental disorders revealed that the proportion of people with substance use disorders who sought treatment in the year of disorder onset ranged from 0.9% to 18.6% and was estimated to increase between 19.8% and 86.1% over 50 years [9]. Although the severity of the illness is associated with the timing of health service use, a study of 84,850 respondents recorded that a maximum of only 46 patients received any care in the previous year [2]. Stigma, geographic location, and distribution of services contribute to fewer people obtaining mental health services [10]. Mental health and addiction services across Canada are associated with long wait times and high cost of assessment of disorders; and inadequate mental health service, stigma, and geographic and demographic inequities act as barriers, hindering individuals with substance use disorders from accessing them [11].

Owing to the COVID-19 pandemic and the associated policies to curb the spread of the virus, many face-to-face addiction services were closed or modified their operational hours, leaving a substantial gap in additional treatments in many jurisdictions [12]. The pandemic thus reduced the support that was available for patients with substance use disorders. The isolation felt by the individuals with substance use disorder also increased the stress, anxiety, and depression experienced by them, which could further worsen their substance abuse [13,14].

Providing health care services through technology during the pandemic was encouraged to reduce the risk of virus transmission and promote the provision of effective treatments [15]. Smartphones, computers, and care delivery via videoconferencing were used more in the pandemic era with reduced physical contact [16,17], and SMS text messaging was also popular during the pandemic [18-23]. Contingent on few psychologists, limited health care services, and high demand for mental health interventions, SMS text messaging as a telehealth intervention has repeatedly been suggested to be beneficial in delivering individualized cognitive behavioral therapy to individuals with mental health and addiction disorders [24,25]. Mobile phone penetration is increasing worldwide, which has increased their use in the health sector in managing health conditions, including substance use disorders [26]. In a study of patients with substance use disorder, approximately 83% of the patients possessed a mobile phone and 86% of the patients were willing to receive their treatment and other interventions via mobile phones [27].

In a review of mobile phone SMS text messaging in clinical and health-related behavioral interventions, 10 out of 16 randomized controlled trials reported a significant improvement in text message interventions compared with usual care [28]. Furthermore, a meta-analysis revealed that web- and computer-based interventions are effective and promising in reducing the stress associated with mental disorders [25]. In contrast, patients’ low motivation for treatment of substance use disorder may contribute to nonadherence to this intervention [4,29]. In addition, a systematic review to characterize the impact of SMS text messaging interventions on people with mental disorders and substance use reported a significant improvement in addiction symptomatology and social function [30]. In a randomized controlled trial in Dublin, Ireland, patients with major depressive disorder and comorbid alcohol use disorder reported more significant cumulative abstinence duration in the intervention group that received twice-daily supportive text messages compared with the control group that received only usual care [31-33]. In the study, 83% of the patients self-reported that the supportive text messages improved their mental health and were a source of motivation for aiding recovery and preventing relapse [31,34]. Similarly, analysis from a randomized controlled trial in Grande Prairie in Alberta, Canada, showed a longer cumulative abstinence duration among patients with alcohol use disorder who received text messages than those who received usual follow-up care after being discharged from a residential treatment program [35].

### Objectives

To the best of our knowledge, no current study has assessed the impact of supportive text messages for addiction care on cravings, anxiety, depression, and recovery during the pandemic. This study aimed to assess the impact of the Text4Hope-Addiction Support program on subscribers. The specific objective of this study was to assess the impact of the program in reducing cravings, anxiety, and depression symptoms in subscribers. Gender is a crucial determinant of mental health [36], and gender analysis aids in improving the understanding of mental health issues and other interventions for mental health [36,37]. Therefore, our interest was also to examine the distribution of all demographic baseline characteristics and isolation conditions based on one of the demographic characteristics (ie, sex at the birth of the participants [male or female]) and to determine any possible significant differences in terms of sex.

## Methods

### Study Design

This was a longitudinal cross-sectional program evaluation with data collected at baseline (on subscription to the Text4Hope-Addiction Support program), at 6 weeks, and after 3 months via web-based survey questionnaires programmed into the REDCap (Research Electronic Data Capture; Vanderbilt University) software hosted at the University of Alberta [38].

### Ethics Approval

This study was approved by the University of Alberta Health Research Ethics Board (approval number Pro00086163). Consent was implied if subscribers completed and returned the survey responses.

### Data Collection

**Figure 1:**
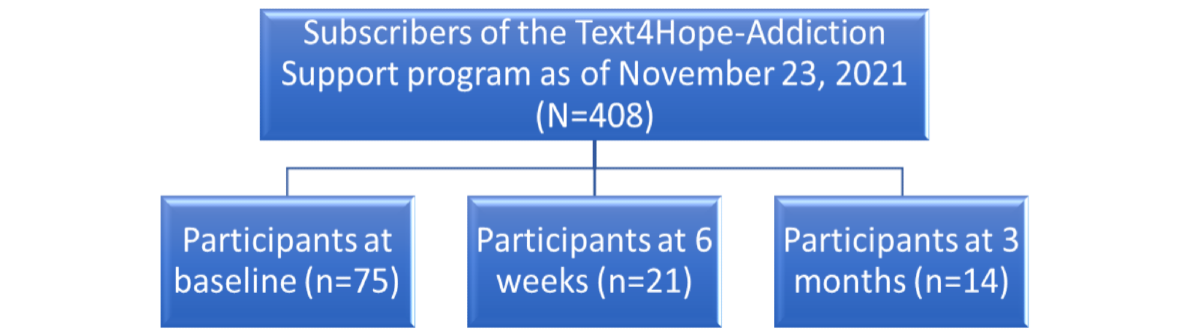
Subscription flowchart.

On enrollment in the program, subscribers received a welcome message that included the link to the baseline web-based survey questionnaire, which captured demographic information (age, gender, employment status, relationship status, ethnicity, and educational level); COVID-19–related self-isolation or quarantine information; and responses on the Brief Substance Craving Scale (BSCS) [39], Generalized Anxiety Disorder-7 (GAD-7) scale [40], and Patient Health Questionnaire-9 (PHQ-9) [41]. The data were collected between July 1, 2020, and November 23, 2021. At 6 weeks (program midpoint) and at 3 months (program end point), individuals received survey links that collected demographic and clinical information similar to those gathered at baseline. During each survey, subscribers provided their cell phone numbers through which they had received text messages, and it was used to link the baseline responses to follow-up responses. The survey took a maximum of 10 minutes to complete, and no incentives were provided to the subscribers. Furthermore, participation in the program was not based on participation in the survey. Figure 1 illustrates the subscriber flowchart and number of subscribers who completed the surveys at each time.

### Outcome Measures

The primary outcome of interest was change in craving score measured from baseline to the 3-month follow-up. The BSCS, a 3-item self-report on a 5-point Likert scale instrument [39] used to assess craving for alcohol and other substances, was used to determine the participants’ intensity, frequency, and length of time cravings. The BSCS consists of the following items: (1) “The INTENSITY of my craving, that is, how much I desired this drug in the past 24 hours;” (2) “The FREQUENCY of my craving, that is, how often I desired this drug in the past 24 hours;” and (3) “The LENGTH of time I spent craving this drug during the past 24 hours.” Each item was rated on a scale of 0 to 4, and the total score of the 3 items was calculated [42].

The secondary outcomes of the study included a change in depression and anxiety scores from baseline to 3 months follow-up time point and included measures related to participants’ satisfaction.

Anxiety symptoms were assessed using GAD-7 [43]. This 7-item screening tool assesses the severity of a likely generalized anxiety disorder. The 7-item inventory asks participants to self-report how frequently they have experienced common anxiety symptomatology over the past month. Ratings are made on a 4-point Likert scale from 0 (not at all) to 3 (nearly every day), with scores ranging from 0 to 21; a score of ≥10 was deemed to indicate likely anxiety, and the higher the score, the more severe the anxiety symptoms [43]. The scores were recategorized into 2 groups (score <10 indicated low anxiety and score ≥10 indicated moderate-to-high anxiety).

The PHQ-9, a 9-item validated tool used to measure the severity of depression in general medical and mental health practice [41], was used to assess depression symptoms in study participants. The questions are scored between 0 (not at all) and 3 (nearly every day). Higher scores on the scale denote more elevated levels of depression [41]. The scale provides a total score 0 to 27. The PHQ-9 scale categorizes depression based on scores: none to minimal (0-4 points), mild (5-9 points), moderate (10-14 points), moderately severe (15-19 points), and severe (20-27 points) [41]. The scores were recategorized as none to mild depression and moderate to severe depression. The reliability and validity of the tool have indicated its good psychometric properties, and the internal consistency of the PHQ-9 is high [41].

The survey contained questions related to participant satisfaction or Text4Hope-Addiction program experience, using the user satisfaction survey from a similar program (Text4Mood program) [44]. This satisfaction scale assessed people’s interest in SMS text messaging and related technological services for health care delivery. Participants were further asked questions regarding their satisfaction with the text addiction program. These questions were categorized into the following 3 categories: (1) perception of how participants coped with stress, anxiety, and depression related to addiction after receiving daily supportive text messages for 3 months. The questions were measured on a 3-point Likert scale: agree, neutral, and disagree. (2) Receptivity of the supportive messages for addiction: these questions were also measured on a 3-point Likert scale: always, often, and sometimes. (3) Recommendation of other technology-based services for future use in addiction recovery support programs: participants chose their response using a 3-point Likert scale (agree, neutral, and disagree).

### Text4Hope-Addiction Support Intervention

Participants self-subscribed to the service by texting “Open2Change” to a short code number. The participants then received unidirectional daily supportive text messages for 3 months. The supportive messages were delivered each day using a computer program at 8 AM MST. The 90 messages built into the program aligned with an addiction counseling and cognitive behavioral therapy framework and were designed to help clients manage cravings, reduce stress and anxiety, minimize depressive symptomology, and improve overall mental health well-being. The messages were written by a clinical psychologist, psychiatrists, and service users and were revised by a multidisciplinary team that included an addiction counselor, a psychiatrist, a mental health therapist, and a service user. An example of a text message is, “Addiction is often cue-based. Identify people, places, and things that trigger the desire to use the drug and avoid them” [4].

### Statistical Analysis

Data were analyzed using SPSS (version 25; IBM Corp) [45]. Demographic, clinical, COVID-19–related variables and other variables were examined against the sex at birth variable using the chi-square or Fisher exact test. Similarly, cross-tabular analyses explored the association of sex at birth with the primary outcome measures using categorical variables for the likelihood that respondents self-reported measures including craving, anxiety, and depressive symptoms during the COVID-19 pandemic. We planned to use a paired sample 2-tailed *t* test to evaluate the change in scores of primary outcome measures from baseline to 6 weeks and 3 months. However, given that only 4/110 (3.6%) subscribers completed the surveys at baseline and at either 6 weeks or 3 months and most (106/110, 96.4%) completed the surveys at only one time point, we modified the analysis as follows.

A 2-tailed independent sample *t* test was conducted initially to examine the difference in mean scores of baseline clinical variables between participants who completed only the baseline survey and those who completed both the baseline and follow-up surveys. No significant differences were observed between the 2 groups. Therefore, the subsequent analysis was run on the 2 independent groups of subscribers after excluding the 4 subscribers who completed the 2-time point surveys. Next, the 3-month scores of the BSCS (craving intensity, craving frequency, and length of time craving drug), PHQ-9, and GAD-7 of subscribers who did not complete baseline surveys were examined against baseline scores of subscribers who did not complete either the 6-week or 3-month surveys, using an independent sample 2-tailed *t* test. When the homogeneity of the variance assumption was violated, Welch *t* test was used instead of the independent sample 2-tailed *t* test. For participants with missing responses for the 3-month time point, missing data were imputed using the last observations carried forward (ie, their 6-week responses) [46].

Satisfaction data were presented as continuous variables measured on a 10-point Likert scale:

(1=very dissatisfied, 5=neutral, and 10=very satisfied). The data were reported as mean and SD. Additional questions related to satisfaction were presented as categorical variables and reported as frequencies and percentages for all study participants. Data are presented using 2-tailed tests, with *P*<.05 as the criterion for significance.

## Results

Of the 408 individuals who subscribed to the Text4Hope-Addiction Support program, 75 (18.4%) participants completed the baseline surveys, for the baseline survey. Of the 408 individuals who subscribed to the program, 97 (23.8%) did not complete the program, yielding a program completion rate of 76.2% (311/408). As illustrated in Figure 1, 5.1% (21/408) of the participants completed the 6-week survey and 3.4% (14/408) completed the 3-month survey, yielding a total sample size of 110 subscribers.

Overall, 33 (30%) males and 77 (70%) females completed at least 1 survey. For the completed surveys, some responses were left blank, which means the total sample size for those individual responses were less than 110. In the overall sample, 40 (37%) individuals were between the ages of 31 and 39 years, 84 (76.4%) individuals were White and 65 (70.7%) individuals had postsecondary education. In addition, 49 (53.3%) were unemployed, 47 (51.1%) were in a relationship, 47 (51.1%) were renting an apartment, and 66 (60%) were not quarantined or self-isolated because of COVID-19. As illustrated in Table 1, the chi-square analysis showed no significant difference in the participants’ sociodemographic characteristics based on sex at birth for the total study sample.

**Table 1:** Distribution of demographic characteristics and isolation conditions based on sex at the birth of the participants (N=110).

Variable^a^						Male (n=33), n (%)		Female (n=77), n (%)		Total, n (%)		Chi-square (*df*)			*P* value	
**Age (years)**													1.61 (2)			.45
	≤30					7 (21.9)		23 (30.3)		30 (27.8)						
	31-39					11 (34.4)		29 (38.2)		40 (37.0)						
	≥40					14 (43.8)		24 (31.6)		38 (35.2)						
**Ethnicity**													4.77 (2)			.09
	White					29 (87.9)		55 (71.4)		84 (76.4)						
	Indigenous					4 (12.1)		14 (18.2)		18 (16.4)						
	Other					0 (0.0)		8 (10.4)		8 (7.3)						
**Education level**													N/A^b^			.56
		<High-school diploma				4 (14.8)		11 (16.9)		15 (16.3)						
		High-school diploma				5 (18.5)		7 (10.8)		12 (13.0)						
		Postsecondary education				18 (66.7)		47 (72.3)		65 (70.7)						
**Employment status**													0.40 (1)			.53
	Employed					14 (51.9)		29 (44.6)		43 (46.7)						
	Unemployed					13 (48.1)		36 (55.4)		49 (53.3)						
**Relationship status**													0.01 (1)			.99
	In a relationship					14 (51.9)		33 (50.8)		47 (51.1)						
	Not in a relationship					13 (48.1)		32 (49.2)		45 (48.9)						
**Housing status**													1.16 (2)			.56
				Own a home		11 (40.7)		19 (29.2)		30 (32.6)						
				Living with family		4 (14.8)		11 (16.9)		15 (16.3)						
				Renting		12 (44.4)		35 (53.8)		47 (51.1)						
**Isolation (quarantine)**													1.85 (1)			.17
			No		23 (69.7)		43 (55.8)		66 (60.0)							
			Yes		10 (30.3)		34 (44.2)		44 (40.0)							

^a^Not all participants provided response.

^b^N/A: not applicable (as Fisher exact test was applied).

Multimedia Appendix 1 illustrates the distribution of clinical and drug-related variables based on the sex at birth of the participants in the study. Of the 75 participants who completed the baseline survey, 45 (60%) participants reported that they had neither received treatment for drug or alcohol in detox nor accessed residential programs. In addition, 16% (12/75) of participants reported that they had overdosed on a recreational drug in the past year; 64.5% (71/110) had no addiction counselor; and 63% (22/35) reported that they had never accessed emergency or crisis services for mental health–related concerns since the start of the COVID-19 pandemic. In addition, of the total 110 participants, 85 (77.3%) participants reported that they did not participate in any narcotics or alcoholics anonymous in-person, via zoom, or on the web during the pandemic. Regarding substance use, 46.4% (51/110) participants indicated that they were on marijuana.

Regarding the self-rated clinical scales, most participants (52/65, 80%) presented with moderate to severe depression, and 70.8% (46/65) presented with moderate to severe anxiety. Craving intensity was either considerable or extreme in 59.1% (42/71) participants. In comparison, 73.2% (52/71) reported craving substances at least several times a day and 67.6% (48/71) reported craving drugs for at least a short time.

As illustrated in Table 2, only 4 participants completed the baseline and 6-week or 3-month surveys. There was no statistically significant difference between the baseline mean scores of PHQ-9, GAD-7, and BSCS for participants who completed both the baseline and either the 6-week or the 3-month surveys and participants who completed only the baseline surveys.

An independent sample 2-tailed *t* test was conducted to compare the BSCS, PHQ-9, and GAD-7 scores for respondents who completed only baseline surveys and those who completed only either the 6-week or 3-month surveys (Table 3).

**Table 2:** Comparison of baseline mean scores of Health Questionnaire-9 (PHQ-9), Generalized Anxiety Disorder-7 (GAD-7), and Brief Substance Craving Scale (BSCS) between participants who completed both baseline and 3-month surveys and those who completed only baseline surveys.

Measure	Scores					*t* test (*df*)		*P* value
	Total number of participants who completed both time points’ surveys	Completed subscribers, mean (SD)	Total number of participants who completed only the baseline survey	Noncompleted subscribers, mean (SD)				
PHQ-9	4	17.25 (7.85)	61	16.85 (7.32)	0.11 (63)		.91	
GAD-7	4	11.50 (8.23)	61	13.80 (6.53)	0.67 (63)		.50	
Craving intensity	4	2.50 (1.00)	67	2.31 (1.38)	0.27 (69)		.79	
Craving frequency	4	2.25 (.50)	67	2.12 (1.27)	0.20 (69)		.84	
Length of time craving drug	4	2.06 (1.32)	67	2.25 (0.96)	0.28 (69)		.78	
Total craving score	4	7.00 (1.83)	64	6.49 (3.62)	0.2 (69)		.78	

**Table 3:** Changes in baseline mean scores of Brief Substance Craving Scale (BSCS), Generalized Anxiety Disorder-7 (GAD-7), and Patient Health Questionnaire-9 (PHQ-9) after the supportive text message intervention (Text4Hope-Addiction).

Measure	Scores						Mean difference (95% CI)		*P* value		*t* test (*df*)		Effect size (Hedges *g*)
	n	Baseline score, mean (SD)	n	3 months score, mean (SD)	Change from baseline (%)								
PHQ-9	61	16.82 (7.32)	23	11.74 (6.21)	30.2	−5.08 (−1.65 to −8.51)		<.01		2.95 (82)		0.72	
GAD-7	61	13.80 (6.53)	23	10.87 (4.58)	21.9	−3.02 (−0.48 to −5.56)		.02		2.38^a^		0.48	
Craving intensity	67	2.31 (1.38)	28	1.36 (1.16)	41.1	−0.95 (−0.37 to −1.55)		<.01		3.21 (93)		0.72	
Craving frequency	67	2.12 (1.27)	28	1.43 (0.96)	32.5	−.69 (−0.16 to −1.22)		.01		2.58 (93)		0.58	
Length of time craving drug	67	2.06 (1.32)	28	1.54 (1.17)	25.2	−0.52 (−0.05 to −1.10)		.07		1.82 (93)		0.41	
Total craving score	67	6.49 (3.62)	28	4.32 (3.04)	33.4	−2.17 (−0.62 to −3.72)		.01		2.79 (93)		0.63	

^a^Welch *t* test was run.

There was a significant difference in craving intensity between the baseline (mean 2.31, SD 1.38) and the 3-month follow-up scores (mean 1.36, SD 1.16; t_93_=3.21; *P*<.01). Craving frequency was also significantly different between the baseline score (mean 2.12, SD 1.27) and the 3-month scores (mean 1.43, SD 0.96; t_93_=2.58; *P*=.01). There was a trend to finding a significant difference in the mean baseline score (mean 2.02, SD 1.32) and 3-month scores (mean 1.54, SD 1.17; t_93_=1.82; *P*=.07) for the length of time craving drug. Finally, the total craving scores at baseline (mean 6.49, SD 3.62) were significantly higher than the 3-month follow-up scores (mean 4.32, SD 3.04; t_93_=2.79; *P*=.01), with a change in score of 33.4% (95% CI −0.62 to −3.72).

There was also a significant difference in depression scores between the baseline (mean 16.83, SD 7.32) and the 3 months follow-up scores (mean 11.74, SD 6.21; t_82_=2.95; *P*<.01). The change between the baseline and 3-month scores was 30.2% (95% CI −1.65 to −8.51).

Similarly, the mean GAD-7 score was significantly lower for participants who received supportive text messages for 3 months (mean 10.78, SD 4.58) than for participants who recorded only baseline scores (mean 13.80, SD 6.53; t_82_=2.38; *P*=.02). The change between the baseline and 3-month scores was 21.9% (95% CI −0.48 to −5.56).

**Program Satisfaction Measures**

The participants rated the supportive text messaging program on a scale of 1 to 10. A score of 1 represents very dissatisfied; 5 represents neutral; and 10 represents very satisfied. The overall mean score for satisfaction was 8.71 (SD 1.33). Approximately 42% (10/24) of participants were very satisfied with the service.

Table 4 reports participant perceptions about the text message addiction program after 3 months of receiving the text message intervention. Overall, 73% (19/26) of participants found the text messages were always positive and affirmative, and 65% (17/26) established that the messages were always relevant and succinct. Most (21/24, 88%) participants reported that they always read the text messages, and 92% (22/24) of participants reported that they at least sometimes returned to read the messages. Overall, 83% (20/24) of participants reported that they took positive or beneficial action after reading the text messages. None of the participants reported reading the messages and taking negative action.

**Table 4:** Participant feedback after 3 months of intervention (N=26).

Feedback		Values, n (%)
**The Text4Hope-Addition text messages were positive.**		
	Always	19 (73)
	Often	7 (27)
**The Text4Hope-Addiction text messages were affirmative.**		
	Always	19 (73)
	Often	7 (27)
**The Text4Hope-Addiction text messages were succinct.**		
	Always	17 (65)
	Often	8 (31)
	Sometimes	1 (4)
**The Text4Hope-Addiction text messages were relevant.**		
	Always	17 (65)
	Often	8 (31)
	Sometimes	1 (4)
**Frequency reading the Text4Hope-Addiction text messages^a^.**		
	Always	21 (87)
	Often	3 (13)
**Action taken after reading text messages^a^.**		
	Read the text and take a positive or beneficial action	20 (83)
	Read the text and took no action	3 (13)
	Other	1 (4)
**Return to read Text4Hope text messages^a^.**		
	Always	5 (21)
	Often	8 (33)
	Sometimes	9 (38)
	Rarely	2 (8)

^a^Not all participants provided response.

Table 5 shows that 88% (23/26) participants agreed that the supportive text messages helped them cope with stress, and 81% (21/26) and 69% (18/26) of participants agreed that the messages helped them cope with anxiety and depression related to their addiction, respectively. In addition, 62% (16/26) of participants reported that the messages helped them cope with addiction-related loneliness. Overall, 85% (22/26) of participants agreed that they felt connected to a support system; 20/26 (77%) were hopeful that they could manage issues related to addiction; and 73% (19/26) of participants agreed the text message intervention helped improve their mental well-being. In comparison, 65% (17/26) of participants agreed that the text messages helped enhance their quality of life.

**Table 5:** Participant opinion on the Text4Hope-Addiction Support program after 12 weeks of intervention (N=26).

Opinion			Values, n (%)
**The daily messages from** **Text4Hope** **have helped me to cope with stress related my addiction.**			
	Agree	23 (88)	
	Neutral	2 (8)	
	Disagree	1 (4)	
**The daily messages from Text4Hope have helped me to cope with anxiety related my addiction.**			
	Agree	21 (81)	
	Neutral	5 (19)	
**The daily messages from Text4Hope have helped me to cope with depression related to my addiction.**			
	Agree	18 (69)	
	Neutral	7 (27)	
	Disagree	1 (4)	
**The daily messages from Text4Hope have helped me to cope with loneliness related to my addiction.**			
	Agree	16 (61)	
	Neutral	7 (27)	
	Disagree	3 (12)	
**The daily messages from Text4Hope have helped me feel connected to a support system.**			
	Agree	22 (84)	
	Neutral	2 (8)	
	Disagree	2 (8)	
**The daily messages from Text4Hope-Addiction have helped me feel hopeful I can manage issues related to my addiction.**			
	Agree	20 (77)	
	Neutral	3 (12)	
	Disagree	3 (12)	
**The daily messages from Text4Hope-Addiction have helped me improve my overall mental well-being.**			
	Agree	19 (73)	
	Neutral	7 (27)	
**The daily messages from Text4Hope-Addiction have helped me enhance my quality of life.**			
	Agree	17 (65)	
	Neutral	7 (27)	
	Disagree	2 (8)	

Table 6 illustrates the participants’ level of agreement with various technology-based services as a component of their health care services to facilitate counseling for addiction, stress, anxiety, and depression. Overall, 87% (13/15) of the participants agreed to recommend web counseling for addiction, stress, anxiety, and depression; 80% (12/15) agreed to recommend telephone counseling for addiction; and 86% (12/14) agreed to recommend telephone counseling for stress, anxiety, and depression. Of the 15 participants, 14 (93%) participants agreed to recommend SMS text messaging to support addiction, and 87% (13/15) participants agreed to recommend text messaging for stress, anxiety, and depression counseling. In addition, 10 (67%) participants recommended email messaging to support addiction care, and 73% (11/15) of participants agreed to recommend email massaging and support for stress, anxiety, and depression. Finally, 80% (12/15) of participants agreed to recommend videoconferencing and telephone conferencing for physical health care, and 87% (13/15) of participants agreed to recommend consultation via telephone conferencing for mental health care.

**Table 6:** Participant recommendations on counseling for addiction, stress, anxiety, and depression (N=15).

Recommendations		Values, n (%)
**Recommend web-based counseling for addiction**		
	Agree	13 (86)
	Disagree	2 (13)
**Recommend web-based counseling for stress, anxiety, and depression**		
	Agree	13 (86)
	Disagree	2 (13)
**Recommend telephone counseling for addiction**		
	Agree	12 (80)
	Neutral	2 (13)
	Disagree	1 (7)
**Recommend telephone counseling for stress, anxiety, and depression^a^**		
	Agree	12 (86)
	Neutral	2 (14)
**Recommend text messaging** **for** **addiction recovery support**		
	Agree	14 (93)
	Neutral	1 (7)
**Recommend text messaging for stress, anxiety, and depression**		
	Agree	13 (87)
	Neutral	2 (13)
**Recommend email messaging for addiction recovery support**		
	Agree	10 (67)
	Neutral	2 (13)
	Disagree	3 (20)
**Recommend email messaging and support for stress, anxiety, and depression**		
	Agree	11 (73)
	Disagree	2 (13)
	Neutral	2 (13)
**Recommend consultation via videoconferencing for mental health care**		
	Agree	13 (87)
	Neutral	1 (7)
	Disagree	1 (7)
**Recommend consultation via videoconferencing for physical health care**		
	Agree	12 (80)
	Neutral	1 (7)
	Disagree	2 (13)
**Recommend consultation via telephone conferencing for mental health care**		
	Agree	13 (87)
	Neutral	1 (7)
	Disagree	1 (7)
**Recommend consultation via telephone conferencing for physical health care**		
	Agree	12 (80)
	Neutral	2 (13)
	Disagree	1 (7)

^a^Not all participants provided response.

## Discussion

### Principal Findings

This study examined the impact of the Text4Hope-Addiction Support program on substance misuse, mental health, and participant satisfaction after they received the text message intervention for 12 weeks. Our study found that 73% (52/71) of subscribers reported cravings for drugs at least several times a day; 72% (51/71) of the participants had moderate to extreme intensity for craving; and for 47% (33/71), the duration of craving drugs was at least somewhat long. The prevalence of moderate to severe depression and anxiety symptoms were 80% and 70.8%, respectively. Owing to the small number of respondents, we used an independent analysis to capture and elicit any difference between baseline and follow-up data among the maximum number of respondents. The analysis revealed a significant improvement in BSCS, PHQ-9, and GAD-7 scores from baseline to 3 months after the intervention. There was a 33.4% difference in the mean total craving score from the baseline. Similarly, the mean depression and anxiety symptom scores saw 30.2% and 21.9% changes (improvement) from baseline to 3 months, respectively. Overall, service satisfaction with the Text4Hope-Addiction Support program was high.

Various reports have indicated that the treatment for substance misuse is geared toward improving relapse and preventing acute episodes [47]. A more innovative, cost-effective, technology-enabled, and easily scalable intervention such as daily supportive SMS text messaging is needed to curb the typically chronic condition of substance misuse [48]. Most participants in this study were female, constituting 70% (77/110) of the total sample. This finding may indicate that women are more likely to seek technology-enabled mental health and additional treatments as observed in other supportive SMS text messaging programs [20,44]. However, it is important to note that sex at birth does not represent the gender identity of participants and may not necessarily affect their responses [49].

Craving intensity yielded the greatest (41.1%) difference in the mean scores at baseline and at 3 months. Our study results are consistent with those of Mason et al [50], who studied students’ readiness to reduce alcohol consumption. The findings from that survey showed that the craving intensity for alcohol was reduced after a month of delivering supportive text messages and there was high participant willingness to minimize alcohol intake. This study found a significant reduction in the frequency of cravings from baseline to 6 weeks (95% CI −0.16 to −1.22). Our findings are consistent with data from a randomized trial that assessed SMS text message interventions among young adults after hospital discharge. After 3 months of delivering text message intervention, participants who received intervention had reduced craving frequency compared with their frequency of carvings at baseline [51].

Analyzing the duration of drug cravings, this study did not find a significant difference between the baseline and 3-month scores of participants. However, given that participants in this study reported reduced craving intensity and frequency at the 3-month time point it is possible that the participants were biased toward reducing the length of time for which they craved drugs. Moreover, the short follow-up time may not have given participants time to explore how long they may stay away from the drug in the absence of an intervention. In contrast, a pilot study reported that after providing supportive text messages to participants with substance misuse, respondents reported that the duration of drug use had significantly reduced in the past month compared with respondents who did not receive the intervention [52]. Moreover, in a randomized controlled trial of supportive text messages for patients with depression and comorbid alcohol use disorder, it was reported that after 3 months of supportive text messaging, participants reported longer days of abstinence of alcohol intake than the control group [34,35]. Suggesting that supportive text messages can reduce alcohol or drug intake and increase the number of days of abstinence.

In this study participants had a 30.2% lower mean depression symptom score at baseline compared with the mean scores at 3 months, and this difference was statistically significant. This finding agrees with other studies that revealed a 24% to 50% improvement in depressive symptomatology in patients with major depressive disorder who received supportive text messages twice daily as part of a randomized controlled trial [33,53]. In addition, a study to assess community attitude toward text messages through a web-based community survey reported that most participants with depression and anxiety symptoms expressed interest in receiving care through their mobile phones [54]. As substance use disorders often coexist with other mental health disorders, such as depression, interventions used to treat addiction may positively impact the treatment of depression symptoms. One study found that addiction treatment for cocaine and alcohol users led to decreased levels of depression as reported by participants [55]. Thus, treatment of substance misuse may improve levels of depression and comorbid substance use disorders in patients.

Clinical practice and research report that anxiety is usually associated with substance use disorder, and its treatment and maintenance are interlaced with those of this comorbidity [56]. Therefore, treatment for anxiety may reduce drug abuse and vice versa. In our study, there was approximately 21.9% reduction from the baseline anxiety symptom scores, which was attributed to the intervention delivered. A similar study showed that anxiety disorders were more strongly related to substance dependence (odds ratio 3.0-6.0) [56]. This study suggests that using adjunctive treatments to target substance use in individuals can be beneficial in minimizing anxiety symptoms.

Participants were very satisfied with the addiction recovery support program, yielding a mean score of 8.71 on a 10-point scale. This result is consistent with other programs that reported an overall satisfaction of 95% [44], which is slightly higher than that reported in the study by Shalaby et al [19,20] that recorded a mean overall participant satisfaction of 8.55 [20]. Participant satisfaction with the program indicates that it is helpful and should be recommended to treat addiction and other related symptoms.

A greater number of participants self-reported their ability to cope with stress related to addiction. This result is consistent with other studies that found that 77.2% of subscribers were able to cope with stress after receiving supportive text messaging [44]. Previous literature has shown that participants are positive about daily supportive text messages [44,57]. Similarly, this result agrees with our study; participants agreed that the messages were positive, affirmative, succinct, and relevant. A survey among young cannabis users concluded that respondents were motivated to reduce their cannabis use after the introduction of supportive text messages, and they reported that the service was generally positive and had a positive impact on their mental health [58]. Supportive text messages have helped people abstain from alcohol and minimize relapse rates [31].

Approximately 85% (22/26) of the participants in this study reported that supportive text messages made them feel connected to the support system. Other studies have reported similar results, where 81% of the participants felt connected to a support system, and 75% felt connected to part of the clinical team (perception data) [20,44,57,59,60].

All (24/24, 100%) the participants reported that they always or often read the text messages, and the proportion is higher than that reported in other studies. In a survey by Agyapong et al [31], 84% of the participants reported reading messages. Similarly, more than three-thirds of the participants returned to read the messages. This is also consistent with a cross-sectional study reporting that 33% of the sample returned to read messages more than once [20]. It is also likely that the content of the text messages, which mental health professionals crafted, contributed to the participants revisiting the messages to help them improve their addictive symptoms. The advantage of text messages is that they allow people to choose when or how often to read them, and individuals have the flexibility to opt in and out of the program at their convenience. This may also contribute to the higher number of individuals returning to read the messages [61]. Most (19/26, 73%) participants in this study reported that the daily supportive text messages improved their overall mental health as did 83% of participants with alcohol use disorder and comorbid depression in another study [31].

There is a crucial demand to develop evidence-based mental health policies that will meet the health needs of individuals, and most prefer to receive such services remotely [62]. Most (14/15, 93%) study participants agreed to recommend SMS text messaging as an intervention to support addiction recovery. Almost three-quarters of the participants agreed to recommend SMS text messaging, email, telephone, or videoconferencing to provide additional treatment support for addiction, stress, anxiety, or depression. Other studies have supported our findings; a randomized controlled trial to explore the perception of students on various interventions for alcohol showed that most participants expressed satisfaction and agreed to recommend SMS text messaging or email interventions to reduce alcohol consumption [49]. Most participants in this study also reported that they would recommend telephone or text messages as their preferred means of receiving addiction treatment. These forms of counseling have an advantage over face-to-face counseling, as they are convenient and provide privacy [58] and can aid health care delivery during pandemics. Isolation and social distancing to minimize the risk of infection in the acute phase affect the number of people accessing mental health service. For example, 63% (22/35) of the participants in this study had never accessed mental health services since the onset of the pandemic.

### Limitations

This study has several limitations, and findings should be put in context when interpreting the results. First, there were low response rates for the baseline and follow-up surveys (6 weeks and 3 months), and only 4 (3%) participants completed the baseline survey and any follow-up survey. Thus, the results presented in the study may not accurately reflect actual changes in scores from baseline to 3 months for participants. Moreover, only 14 participants completed the 3-month surveys [60,63]. For 21 participants, the survey responses for the outcome measures at 3 months were imputed from their 6-week survey responses. Therefore, it is likely that for participants for whom there was data imputation, the 6-week outcome data may not accurately reflect their clinical outcomes at 3 months. In addition, as the study design and analysis plan were constrained by the lack of participant completion at both the baseline and follow-up surveys, it was impossible to control for baseline variables, as these variables did not exist for the intervention group. Furthermore, the supportive text messages were delivered for 3 months, with outcome measures evaluated at the end point of 3 months. Therefore, the long-term impact of this intervention cannot be determined from this study. In addition, the study participants may not represent individuals with addiction and may not be generalizable to a clinical sample of patients with addiction. In addition, although the BSCS, a 3-item scale, has adequate reliability, interitem consistency, and face validity, the brevity and narrow focus of the dimensions that were assessed diminish the construct and concurrent validity of this scale [42].

The study found no statistically significant difference between the baseline mean scores of PHQ-9, GAD-7, and BSCS for participants who completed both the baseline and either the 6- week or the 3-month survey and those who completed only the baseline surveys. The loss to follow-up surveys may be attributed to other adverse effects such as continuous use of substances after completing the baseline survey and loss to follow-up to document the progress.

Finally, participants’ feedback and perception of their ability to cope with stress, anxiety, and depression associated with drug addiction were self-reported and not assessed clinically, although standardized screening scales were used. Notwithstanding these limitations, the findings from this study indicate that supportive text messages are promising and have the potential to reduce craving symptoms, depression, and anxiety in drug addictions.

### Conclusions

The study’s findings suggest that craving, anxiety, and depression symptoms were high among subscribers of Text4Hope-Addiction Support program during the pandemic, and supportive text message intervention could help curtail craving, depression, and anxiety symptoms experienced by individuals with drug addiction. Clinicians and policy makers may find it beneficial to incorporate supportive text messages to supplement addiction treatment and improve client outcomes. Further studies are needed to investigate other effects of text message interventions on drug addiction.

## Appendix

Multimedia Appendix 1 Distribution of clinical and drug-related variables based on sex at birth of the participants.

## Abbreviations

BSCS: Brief Substance Craving Scale
GAD-7: Generalized Anxiety Disorder-7
PHQ-9: Patient Health Questionnaire-9
REDCap: Research Electronic Data Capture
