# Supplementary material for: Effects of Text4Hope-Addiction Support Program on Cravings and Mental Health Symptoms: Results of a Longitudinal Cross-sectional Study
Source: JMIR Form Res. 2023 Mar 1;7:e40440. doi: 10.2196/40440 (PMC10018381; doi:10.2196/40440)
Supplement: Multimedia Appendix 1 [file formative_v7i1e40440_app1.docx]

**Distribution of clinical and drug-related variables based on sex at birth of the participants (N=110).**

| **Variables** | **Male, n (%)** | | **Female, n (%)** | | **Total, n (%)** | **Chi-square** | ***P* value** |
| --- | --- | --- | --- | --- | --- | --- | --- |
| **Drug or alcohol treatment or detox program** | | | | | | 1.30 | .26 |
| Yes | 11 (50.0) | | 19 (35.8) | | 30 (40.0) |  |  |
| No | 11 (50.0) | | 34 (64.2) | | 45 (60.0) |  |  |
| **Residential treatment program** | | | | | | 2.74 | .10 |
| Yes | 12 (54.5) | | 18 (34.0) | | 30 (40.0) |  |  |
| No | 10 (45.5) | | 35 (66.0) | | 45 (60.0) |  |  |
| **Past year overdose (recreational drug)** | | | | | | 0.11 | .74 |
| Yes | | 4 (18.2) | | 8 (15.1) | 12 (16.0) |  |  |
| No | | 18 (81.8) | 45 (84.9) | | 63 (84.0) |  |  |
| **Have addiction counselor** | | | | | | 0.02 | .90 |
| Yes | 12 (36.4) | | 27 (35.1) | | 39 (35.5) |  |  |
| No | 21 (63.6) | | 50 (64.9) | | 71 (64.5) |  |  |
| **Frequency of mental health service visits during the pandemic** | | | | | | 0.63 | .77 |
| Not at all | 8 (72.7) | | 14 (58.3) | | 22 (62.9) |  |  |
| Once | 1 (9.1) | | 4 (16.7) | | 5 (14.3) |  |  |
| More than once | 2 (18.2) | | 6 (25.0) | | 8 (22.9) |  |  |
| **In-person AA^a^ or NA^b^ meeting** | | | | | | 0.01 | .91 |
| Yes | 5 (15.2) | | 11 (14.3) | | 16 (14.5) |  |  |
| No | 28 (84.8) | | 66 (85.7) | | 94 (85.5) |  |  |
| **Zoom AA or NA meeting** | | | | | | 0.81 | .37 |
| Yes | 7 (21.2) | | 11 (14.3) | | 18 (16.4) |  |  |
| No | 26 (78.8) | | 66 (85.7) | | 92 (83.6) |  |  |
| **Online AA or NA meeting** | | | | | | 2.10 | .15 |
| Yes | 5 (15.2) | | 5 (6.5) | | 10 (9.1) |  |  |
| No | 28 (84.8) | | 72 (93.5) | | 100 (90.9) |  |  |
| **Participated in AA or NA meeting** | | | | | | 0.56 | .46 |
| Yes | 9 (27.3) | | 16 (20.8) | | 25 (22.7) |  |  |
| No | 24 (72.7) | | 61 (79.2) | | 85 (77.3) |  |  |
| **On psychotropic medications** | | | | | | | |
| Downers or sedatives | 2 (6.1) | | 6 (7.8) | | 8 (7.3) | 0.10 | .75 |
| Benzos | 3 (9.1) | | 8 (10.4) | | 11 (10.0) | 0.04 | .84 |
| Hallucinogens | 4 (12.1) | | 7 (9.1) | | 11 (10.0) | 0.24 | .63 |
| Alcohol | 10 (30.3) | | 40 (51.9) | | 50 (45.5) | 4.37 | .04 |
| Solvents or inhalants | 0 (0) | | 2 (2.6) | | 2 (1.8) | 0.87 | .35 |
| Heroin or other opiates | 1 (3.0) | | 4 (5.2) | | 5 (4.5) | N/A^c^ | .99 |
| Marijuana | 11 (33.3) | | 40 (51.9) | | 51 (46.4) | 3.22 | .07 |
| GHB^d^ | 2 (6.1) | | 4 (5.2) | | 6 (5.5) | 0.03 | .86 |
| Anabolic steroids | 0 (0) | | 1 (1.3) | | 1 (0.9) | N/A^c^ | .99 |
| Stimulants | 15 (45.5) | | 27 (35.1) | | 42 (38.2) | 1.06 | .30 |
| **PHQ-9^e^ scale—baseline** | | | | | | 0.67 | .50 |
| At most mild depression | 5 (26.3) | | 8 (17.4) | | 13 (20.0) |  |  |
| Moderate to severe depression | 14 (73.7) | | 38 (82.6) | | 52 (80.0) |  |  |
| **GAD-7^f^—baseline** | | | | | | 0.75 | .39 |
| None to mild anxiety | 7 (36.8) | | 12 (26.1) | | 19 (29.2) |  |  |
| Moderate to severe anxiety | 12 (63.2) | | 34 (73.9) | | 46 (70.8) |  |  |
| **Craving intensity—baseline** | | | | | | N/A^c^ | .19 |
| None at all | 7 (33.3) | | 5 (10.0) | | 12 (16.9) |  |  |
| Slight | 2 (9.5) | | 6 (12.0) | | 8 (11.3) |  |  |
| Moderate | 3 (14.3) | | 6 (12.0) | | 9 (12.7) |  |  |
| Considerable | 7 (33.3) | | 22 (44.0) | | 29 (40.8) |  |  |
| Extreme | 2 (9.5) | | 11 (22.0) | | 13 (18.3) |  |  |
| **Craving frequency—baseline** | | | | | | N/A^c^ | .32 |
| Never | 2 (9.5) | | 7 (14.0) | | 9 (12.7) |  |  |
| Almost never | 6 (28.6) | | 4 (8.0) | | 10 (14.1) |  |  |
| Several times | 7 (33.3) | | 21 (42.0) | | 28 (39.4) |  |  |
| Regularly | 3 (14.3) | | 8 (16.0) | | 11 (15.5) |  |  |
| Almost constantly | 3 (14.3) | | 10 (20.0) | | 13 (18.3) |  |  |
| **Length of time craving drug—baseline** | | | | | | N/A^c^ | .58 |
| None at all | 5 (23.8) | | 8 (16.0) | | 13 (18.3) |  |  |
| Very short | 4 (19.0) | | 6 (12.0) | | 10 (14.1) |  |  |
| Short | 3 (14.3) | | 12 (24.0) | | 15 (21.1) |  |  |
| Somewhat long | 8 (38.1) | | 17 (34.0) | | 25 (35.2) |  |  |
| Very long | 1 (4.8) | | 7 (14.0) | | 8 (11.3) |  |  |

^a^AA: alcoholics anonymous.

^b^NA: narcotics anonymous.

^c^N/A: not applicable as Fisher exact test was applied.

^d^GHB: Gamma-hydroxybutyric acid

^e^PHQ-9: Patient Health Questionnaire-9.

^f^GAD-7: Generalized Anxiety Disorder-7.
